# Apoptosis during ZIKA Virus Infection: Too Soon or Too Late?

**DOI:** 10.3390/ijms23031287

**Published:** 2022-01-24

**Authors:** Jonathan Turpin, Daed El Safadi, Grégorie Lebeau, Morgane Krejbich, Camille Chatelain, Philippe Desprès, Wildriss Viranaïcken, Pascale Krejbich-Trotot

**Affiliations:** 1 Processus Infectieux en Milieu Insulaire Tropical (PIMIT), Université de La Réunion, INSERM UMR 1187, CNRS 9192, IRD 249, Plateforme CYROI, 97490 Sainte-Clotilde, France; jonathan.turpin@univ-reunion.fr (J.T.); daedalsafadi@gmail.com (D.E.S.); gregorie.lebeau@univ-reunion.fr (G.L.); philippe.despres@univ-reunion.fr (P.D.); wildriss.viranaicken@univ-reunion.fr (W.V.); 2 CRCI2NA, Centre de Recherche en Cancérologie et Immunologie, Université de Nantes, Université d’Angers, INSERM UMR 1307, 44000 Nantes, France; morgane.krejbich@etu.univ-nantes.fr (M.K.); camille.chatelain@etu.univ-nantes.fr (C.C.)

**Keywords:** apoptosis, cell death, Zika virus, ZIKV

## Abstract

Cell death by apoptosis is a major cellular response in the control of tissue homeostasis and as a defense mechanism in the case of cellular aggression such as an infection. Cell self-destruction is part of antiviral responses, aimed at limiting the spread of a virus. Although it may contribute to the deleterious effects in infectious pathology, apoptosis remains a key mechanism for viral clearance and the resolution of infection. The control mechanisms of cell death processes by viruses have been extensively studied. Apoptosis can be triggered by different viral determinants through different pathways as a result of virally induced cell stresses and innate immune responses. Zika virus (ZIKV) induces Zika disease in humans, which has caused severe neurological forms, birth defects, and microcephaly in newborns during the last epidemics. ZIKV also surprised by revealing an ability to persist in the genital tract and in semen, thus being sexually transmitted. Mechanisms of diverting antiviral responses such as the interferon response, the role of cytopathic effects and apoptosis in the etiology of the disease have been widely studied and debated. In this review, we examined the interplay between ZIKV infection of different cell types and apoptosis and how the virus deals with this cellular response. We illustrate a duality in the effects of ZIKV-controlled apoptosis, depending on whether it occurs too early or too late, respectively, in neuropathogenesis, or in long-term viral persistence. We further discuss a prospective role for apoptosis in ZIKV-related therapies, and the use of ZIKV as an oncolytic agent.

## 1. Introduction

The recent COVID pandemic reminds us of how vulnerable the world’s population is to zoonotic RNA viruses of medical concern. Between 2007 and 2016, the emergence of epidemic strains of mosquito-borne Zika virus (ZIKV), a member of the flavivirus genus of *Flaviviridae* family, was in the spotlight. [1]. At that time, the rapid expansion of the Zika epidemic throughout the intertropical zone had already received international attention. Health authorities from several countries were concerned and had to deal with a disease whose clinical outcomes such as congenital syndromes and microcephaly in infants were more severe and frequent than previously known [2]. The international scientific community had to react to a poorly documented infectious agent whose interactions with its hosts (human or mosquitoes), pathogenesis, and unusual transmission pathways had been only partially or not described [3].

The pathogenicity of an infectious agent depends largely on the cytopathic effects it will produce in its target cells. In contrast, programmed cell death (PCD) is an urgent and necessary defense response, implemented by the infected host to disrupt virus multiplication and propagation, thereby preserving other cells and surrounding tissue [4]. The death of infected cells by apoptosis also plays a crucial role in promoting antigen presentation and recruiting immune cells, thus facilitating the infection resolution [5,6]. Failures in viral clearance are widely attributed to defects in the infected cells’ auto-destruction [7].

Despite the tissue damage and the subsequent critical loss of cell populations, PCD by apoptosis is thus considered a ‘necessary evil’ of the body’s response in its efforts to get rid of the infectious agent.

In the case of ZIKV infection and pathogenesis, massive apoptosis of neuronal progenitor cells (NPCs), early in fetal development, has been proposed as an explanation for the specific induced central nervous system (CNS) disorders in newborns and infants [8,9]. At the same time, the finding that ZIKV can persist in adults in the urinary and reproductive tracts and semen, for many months after the acute infection episode, is an indication that viral clearance may be incomplete and associated with impaired or delayed apoptosis [10].

In this review, we propose examining the available data on ZIKV-induced apoptosis and the state of knowledge regarding the virus’s ability to manipulate, delay, or inhibit the cell death response. We also discuss antiviral therapies based on the remediation of apoptosis, and given its unique dual relationship with cell death, the possibility of using ZIKV as an oncolytic agent.

## 2. Apoptotic Cell Death

### 2.1. Features of Apoptosis

**Figure 1:**
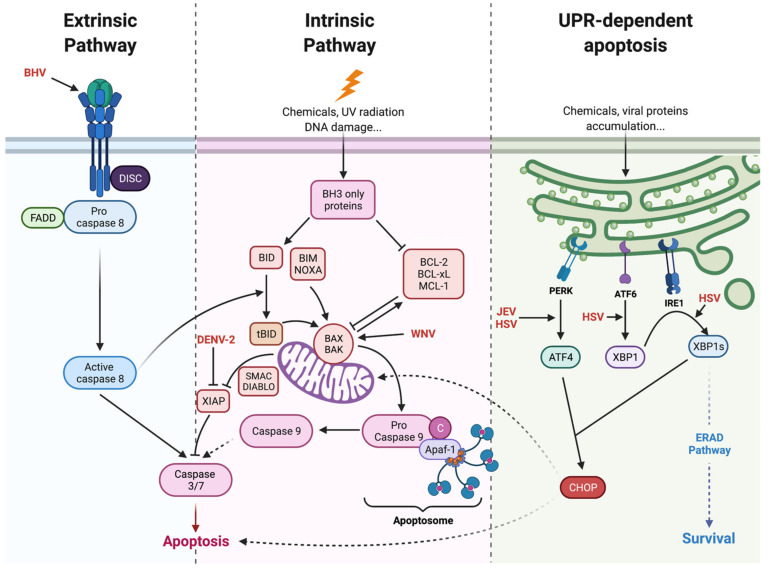
The different pathways of apoptosis. Apoptosis can follow several pathways, depending on the signals integrated by the cell and the respective influence of the mobilized pro- and anti-apoptotic factors. The extrinsic pathway is induced by external death signals such as TNFα and mediated by death receptors (FAS, DR4 expressed on the cell surface. The intrinsic pathway is induced by internal stimuli such as DNA damage, oxidative stress, or intracellular parasites. Apoptosis could also be activated by unresolved endoplasmic reticulum stress (ER stress) and unfolded protein response (UPR) through C/EBP HOmologous Protein (CHOP) [15,16,17,18,19,20,21,22]. Viruses such as BHV, DENV, WNV, JEV, and HSV (in red) can induce apoptosis by the activation of different pathways [23,24,25,26,27,28,29,30,31,32,33,34,35].

Apoptosis is one of the genetically programmed cell deaths that regulates tissue homeostasis and aims to eliminate non-viable, stressed, injured, or infected cells [11]. Cell death by apoptosis is a process defined by canonical morphological and molecular criteria. Apoptosis induces specific morphological changes that lead to cell implosion and disappearance. This is supported by the condensation and fragmentation of the nucleus (pyknosis and karyorrhexis), followed by plasma membrane blebbing and cell disassembly [12]. The process ends by efferocytosis, with cell residues such as blebs or apoptotic bodies being engulfed by non-dying neighboring cells or professional phagocytes [13]. At the molecular level, the caspase family of proteases is required for the completion of the operation, which entails initiation and execution phases [14]. Mitochondria plays a major role in the initiation of apoptosis through the permeabilization of its outer membrane (MOMP) [15]. The members of the BCL-2 protein family are the main actors of the MOMP (Figure 1). Among the family are proteins with the BH3 domain only (e.g., BID, BIM, NOXA, BAD), which activate or control other BCL-2 proteins (the pro-apoptotic BAX and BAK) to initiate the complex formation required for MOMP, while anti-apoptotic regulators such as BCL-2, BCL-XL, and MCL-1 inhibit its formation [16]. MOMP leads to the release of cytochrome-c from the mitochondria and the activation of the protein apoptosis protease activating factor 1 (APAF-1). Oligomerized cytochrome-c-APAF-1, together with pro-caspase-9, form a macromolecular complex called apoptosome [17]. Activated caspase-9 from the apoptosome complex will then process the executioner pro-caspase-3 into active caspase-3, which in turn degrades numerous substrates such as poly ADP-ribose polymerase (PARP) [18].

Several typical situations are capable of initiating apoptosis (Figure 1). Receiving death signals activates the extrinsic pathway through TNF receptors (TNFR) and Fas receptors (CD95) and leads to death-inducing signaling complex (DISC) formation and activation of caspase-8. This initiating caspase can activate BID, which will then activate the mitochondrial pathway [19,20]. Cell stresses decompensations, damages of physico-chemical origin (UV, drugs, reactive oxygen species) or following endoplasmic reticulum (ER) stress, and an unresolved unfolded protein response (UPR), lead to an intrinsic pathway of apoptosis with mitochondrial membrane modifications and caspase-9 activation [21,22].

### 2.2. Virus-Induced Apoptosis

Each stage of a viral infection cycle is likely to drive pro-apoptotic signaling. First, some viruses can trigger an extrinsic pathway, just upon attachment and entry. In the cases reported in the literature (e.g., for the bovine herpesvirus (BHV),) functional proximity between the viral cell surface binding site and death receptors seems to be involved [23]. Second, intrinsic pro-apoptotic signaling in infected cells is the logical consequence of the multiple decompensations caused by viral multiplication on cellular homeostasis. It is argued that to ensure its replication cycle and the production of its progeny, the virus robs the cell of its resources for its own benefit. This hijacking of cellular metabolism, the diversion of protein synthesis, and nucleotide pools have been described as being able to disrupt mitochondrial homeostasis and activate the intrinsic pathway of apoptosis [24,25,26].

In addition to metabolic reprogramming, the increasing amounts of viral compounds following virus replication are likely to generate stresses that lead to apoptosis initiation. The considerable input of proteins to be processed by the endoplasmic reticulum leads to ER stress and UPR. This adaptive response is implemented by cells for its antiviral effects at the end. UPR is a way to reduce viral protein synthesis and ultimately results in the self-destruction of the cell, thus bypassing the virus. Many viruses have replication cycles that lead to UPR-dependent apoptosis. This kind of viral induced cellular stress, followed by cell death has been extensively documented, for example, in Japanese encephalitis virus (JEV) and herpes simplex virus (HSV) infections [27,28,29]. Viral compounds have been identified as capable of inducing apoptosis on their own. Among the flaviviruses, the overexpression of viral proteins, both of structural and non-structural origin, or of peptides derived from these proteins such as the peptide called apoptoM has demonstrated their cytotoxic capacity [30,31,32,33]. Unfortunately, the exact mechanisms of their pro-apoptotic action remain poorly understood. One assumption is that some of them may be more specifically responsible for the UPR response by initiating the formation of misfolded aggregates in the ER lumen.

The literature on virus-induced apoptosis frequently reports that viruses can change the dominant relationship between pro- and anti-apoptotic factors. It has been shown that Dengue virus (DENV2) induces apoptosis via the expression of XIAP associated factor 1 (XAF1), an inhibitor of the anti-caspase activity of the XIAP protein [34]. Additionally, several viruses are known to upregulate the expression of pro-apoptotic factors. West Nile virus (WNV), for example, induces apoptosis via increased transcription of the pro-apoptotic factor BAX [35] (Figure 1).

Furthermore, it is increasingly suggested that pattern recognition receptor (PRR) activation by viral compounds, in particular viral genomes in all their forms (DNA, cDNA, single-, and double-stranded RNA) and the subsequent innate immunity signaling, can result in apoptosis [36]. The mitochondrial antiviral-signaling protein (MAVS) recruitment by RIG-I and MDA5 sensing can disrupt the mitochondrial membrane potential and lead to caspase activation [37]. IRF3, an interferon response factor, can directly promote BAX oligomerization at the outer mitochondrial membrane or act as a transcription factor able to upregulate the expression of BH3-only NOXA [38,39]. Of note, activation of PRRs, in addition to promoting the immune response with inflammatory cytokine production, seems to lead to various forms of cell death such as pyroptosis and necroptosis [40]. This diversity of signaling pathways in response to viral infections has led to the emergence of a PANoptosis concept, based on the crosstalk between key molecules of each type of PCD [41].

## 3. Zika Infection

### 3.1. Zika Virus, Historical Data

Zika virus (ZIKV) is a pathogenic arbovirus, vectorized by mosquitoes of the genus *Aedes* (*Ae.*) [42]. It belongs to the *Flaviviridae* family and the *Flavivirus* genus such as DENV, WNV, and many others. It was initially isolated in 1947 from a sentinel rhesus monkey during a study on the circulation of the Yellow Fever virus (YFV) in Uganda and found later in *Ae. Africanus* mosquitoes [43]. The first human case was described in 1952 [44], but little research on ZIKA arbovirosis has been conducted over the past century as only a few outbreaks and sporadic cases have been reported, with only mild characteristics evoked for the symptoms. This has changed over the last twenty years with the re-emergence of ZIKV, associated with major epidemics and severe forms of ZIKA diseases. ZIKV returned to the spotlight in 2007 in Micronesia [45], then the virus reached French Polynesia in 2013 [46], and affected Brazil in 2015, where an estimated 1.5M people were infected [47]. This epidemic emergence led the World Health Organization (WHO) to declare ZIKA disease as a public health emergency in February 2016. Analysis of circulating viruses revealed the existence of two lineages, an African lineage and an Asian-American lineage containing all the strains that have led to the epidemic outbreaks [48]. Today, the virus circulates endemically in several regions and outbreaks are occasionally observed such as in Jaipur in India [49]. Further emergences could lead to sporadic outbreaks in certain places described as being at high risk of introduction [50,51].

### 3.2. Zika Virus Transmission and Clinical Outcomes

Associated with the virus’s emergence, new modes of transmission have been discovered. Several hypotheses have been put forward to explain the extent and rapid expansions of the last outbreaks. Among them was the switch from historical African and sylvatic species of mosquitoes to more urban vectors, mainly *Ae. Aegypti*, but also *Ae. Albopictus*, *Ae. Polynesiensis*, *Ae. Vittatus*, *Ae. Unilineatus*, and *Ae. Hensilli* [52]. More unusual among flaviviruses, non-vector modes of transmission have been reported for ZIKV such as sexual transmission. This has led the Centers for Disease Control (CDC) and the WHO to recommend the use of condoms or avoidance of sex for at three months [53,54]. Maternal–fetal vertical transmissions were also reported during pregnancy [55].

Clinical forms are in most cases common to many arbovirosis and include fever, rash, arthralgia, and sometimes conjunctivitis due to direct eye infection [56]. The febrile state usually resolves in less than a week [45].

However, serious complications have occurred after infection with ZIKV. The most outstanding ones have been neurodevelopmental injuries during mother-to-fetus transmission, but peripheral nervous system disorders such as Guillain-Barré syndrome in adults have also alerted the international scientific community. High levels of anti-ganglioside antibodies in affected patients with this syndrome may be linked to this pathology [57,58].

Numerous cases of congenital Zika syndrome (CZS) including microcephaly due to arrested development of the cerebral cortex [59], brain calcifications, intrauterine growth restriction, and fetal death were reported during the 2013 Polynesian and 2015 Brazilian epidemics and among imported cases all over the world [9,59]. The case fatality rate for microcephalic infants is estimated to be around 10%, with the worst outcomes seen in late preterm and/or low birth weight newborns [60,61].

Another surprising feature of Zika infection was the discovery that viral RNA could be found until 40 days in serum and until 120 days in semen after the onset of symptoms [62,63]. Moreover, a recent report suggests that viremia could be found three years after infection, probably due to genetic susceptibility [64]. All these data suggest that ZIKV can persist in the host body after the acute phase of the viral infection.

### 3.3. Zika Virus Structure and Life Cycle

**Figure 2:**
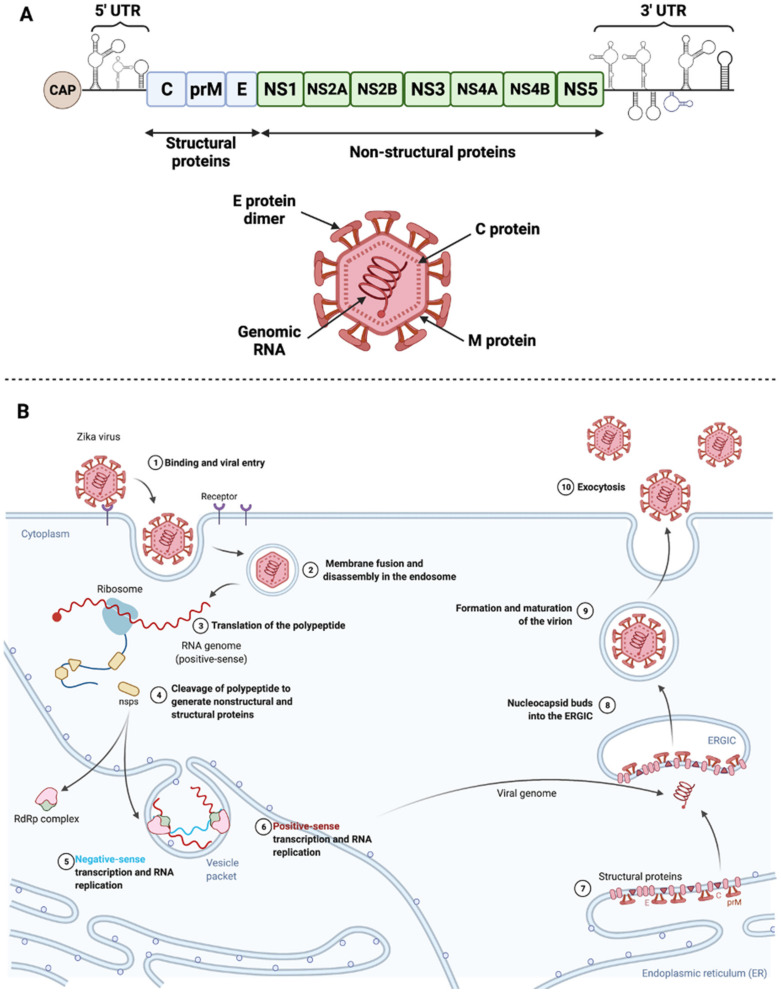
Zika virus structure and life cycle. (**A**) Scheme of Zika virus genome and structure. Zika virus genome encodes 10 viral proteins, three structural, and seven non-structural. 5′ and 3′ UTR refer to untranslated regions [65]. (**B**) ZIKV binds its specific receptors and is subsequently internalized into the target cell via clathrin-mediated endocytosis (1). Viral RNA is released into the cytoplasm following virus fusion with the endosomal membrane and capsid disassembly (2). Upon release, positive-sense RNA is translated into a polypeptide incorporated in the endoplasmic reticulum (ER) membrane and cleaved by proteases into structural and non-structural proteins (3,4). It will also be transcribed into negative-sense RNA that will serve as a template for RNA replication that takes place in ER vesicle packets (5,6). Viral proteins are accumulated and structured at the level of the ER (7). Virus assembly, budding, and maturation then occur in the ER-Golgi intermediate compartment (ERGIC) and in the Golgi apparatus, respectively (8,9). New viruses are released by exocytosis (10). Adapted from Lebeau et al. [66].

The ZIKV genome is composed of a positive 11 kb single-stranded RNA that encodes 10 proteins. Among these proteins, three are structural (C, prM, E) and seven are non-structural (NS1, NS2A/B, NS3, NS4A/B, NS5) as shown in Figure 2A and reviewed by Petersen et al. [65]. In classical vector transmission, an infected mosquito will deliver the virus to the epidermis of the host during its blood meal. ZIKV will further enter its target cells and move along the endocytic pathway to reach the endosome. Fusion of the viral envelope and decapsidation will release the viral genome into the cytoplasm where it will be directly translated into a polyprotein further cleaved by viral proteases (NS2B/NS3) and host proteases of the endoplasmic reticulum (ER) and Golgi apparatus. The envelope protein (E) is involved in virus entry and receptor recognition. The membrane (prM) protein present in the immature virion prevents premature fusion during egress; it is further cleaved in M protein. The nucleocapsid protein (C) holds the viral RNA. The non-structural proteins participate mainly in viral replication. The NS5 protein implements genome replication platforms at the ER membrane, with cofactor proteins such as NS1, NS4A, and NS4B. NS1 is a singular flaviviral protein that can be secreted and found circulating in the bloodstream. Its role in Zika disease is not clearly identified, but it is known to be highly immunogenic and potentially involved in metabolic modifications [66]. After incorporation of neo-synthesized viral genomes in capsids, and budding from the ER membrane, virions move to the Golgi apparatus where the host furins lead to a mature virion. The non-coding regions of the viral RNA (3′UTR), which leads to the production of small flavivirus RNA (sfRNA) that accumulate in infected cells, are known to be important in modulating cell responses [67].

### 3.4. Zika Virus Tropism and Pathogenesis

Although not classified as an encephalitic flavivirus, ZIKV has been responsible for debilitating CNS disorders in neonates in the severe forms of infection described from 2013 in Polynesia and 2015 mainly in Brazil [68]. Most of the documented CZS mention hypoplasia of the cerebellum, delayed myelination, calcifications, and many cortical abnormalities [69]. ZIKV infection during the first or second trimesters of pregnancy also resulted in hearing loss and blindness in newborns [70]. In-vitro, ex-vivo, and in-vivo study models have been developed to understand the pathological processes behind these clinical outcomes.

Multiple strains of ZIKV of African or Asian-American origin have been isolated and used for the study of ZIKV tropism and pathogenesis. Some studies have used recent epidemic clinical isolates and other infectious clones for the analysis of (i) viral tropism and (ii) molecular determinants of pathogenesis [71]. Some teams [72,73] have reported in their reviews that ZIKV infection in mouse models (neonatal wild-type mice, adult immunocompromised Ifnar1^−/−^ mice) had reproduced the main features of the human disease including infection of the placenta and neuro-invasiveness, leading to reduced brain development and fetus size. Many studies have shown that in these models, the virus could accumulate in various organs such as the brain with associated damages, in the spinal cord, in eye associated tissues, male and female reproductive tracts, gonads, and kidneys. Neurotropic pathogenesis has been attributed to the depletion of neural progenitor cells; however, their infection does not appear to be the only cause of their loss [74,75,76]. In non-human primate models, infection also resulted in fetal brain lesions and ZIKV RNA was found in saliva and seminal fluids for at least three weeks after the end of viremia [76].

In the field of in-vitro models to investigate the interaction of the virus with its human host, Marazzo and colleagues recently reviewed how the development of neurosphere organoid cultures has provided insights into the specific neuropathological mechanisms of ZIKV [77]. The different studies that have been carried out using these models have confirmed that neuronal and neural progenitor cells were susceptible to ZIKV [78].

Finally, ZIKV was shown able, in-vitro and in-vivo, to infect trophoblasts, fetal endothelial cells of the placenta, placental macrophages (Hofbauer cells), and perivascular cells [79,80]. It has also been shown to infect a variety of cultured cells and cell lines of both fibroblast and epithelial origin [81].

## 4. Zika Virus and Apoptosis

**Figure 3:**
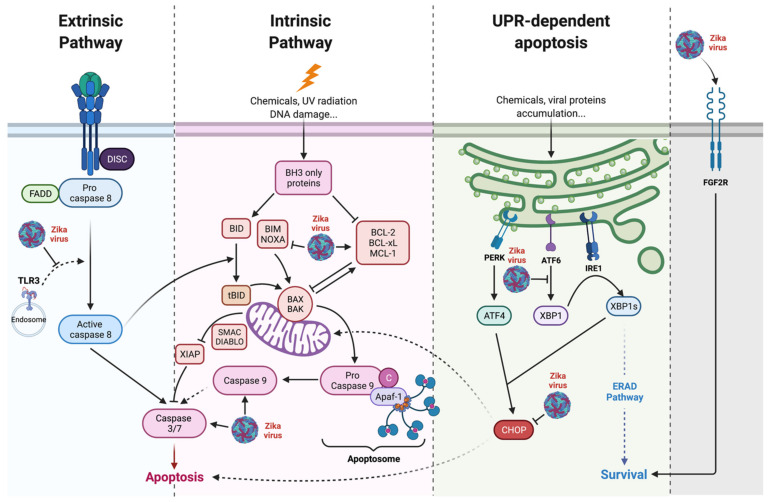
Zika virus controls apoptosis. ZIKV acts on anti-apoptotic factors such as Bcl-2 or Bcl-xL mainly through an effect mediated by the non-structural proteins. ZIKV controls CHOP activity during UPR, limiting its pro-apoptotic activity [92]. DsRNA/TLR3 induced apoptosis is controlled by ZIKV through NS2B/3 [82]. ZIKV also stimulates the expression of pro survival factors such as fibroblast growth factor 2 (FGF2) [88,93].

As the cytopathic activity of the virus may explain the pathogenic processes and some clinical outcomes, many studies have evaluated the viral-induced forms of PCD in in-cellulo, ex-vivo, in-vivo, and in animal models of ZIKV infection. The onset timing of cytopathic effects and the search for markers specifying the virally mediated cell death were conducted and compared to the course of infection and completion of the viral cycle. Various apoptosis data obtained for different ZIKV strains and infection models have been compiled in Table 1. Hence, the main protagonists of apoptosis that are under the control of ZIKV are highlighted in Figure 3.

**Table 1:** Apoptotic induction in different biological models infected by ZIKV.

Virus Strain	Cellular Model	Apoptotic Cues	Apoptosis and ZIKV Infection Interplay	Reference
MR766: East African strain isolated from sentinel rhesus in Uganda in 1947 (Dick, Kitchen, and Haddow 1952)	HT1080 (Human epithelial cells derived from fibrosarcoma)	Apoptotic cell death markers were cleavage of Caspase 3 and PARP.	Apoptosis was shown to be delayed by inhibition of the JAK-STAT pathway by ZIKV NS2B/3 proteins.	[82]
Brazil 2015 (KU940228) Brazilian strain 2015 (Calvet et al., 2016)	hNPCs (Human Neural Progenitor cells)	Early apoptosis was induced at 24 h.p.i. with caspase 8, 9, and 3 activation. This viral strain was found to be highly deleterious to human neural progenitor cells.	Apoptosis was shown to limit viral production. This was reversed by the use of Z-VAD which induced an increase in intracellular viral RNA.	[83]
MR766	hNPCs	Apoptotic cell death was induced at 72 h.p.i. Caspase 3 expression was highly increased 3 d.p.i.	N.D.	[84]
cDNA encoding the E, prM-E and M-E proteins of the Haitian ZIKV strain (KU509998.3) isolated in 2014 (Lednicky et al., 2016)	PC12 cells (Rat pheochromocytoma cells)	Intrinsic mitochondrial pathway.	Envelope viral protein induces apoptosis by increasing BAX expression and decreasing Bcl-2 expression at the transcriptional and translational levels at 48 h.p.t.	[85]
H/PF/2013: French Polynesian 2013 clinical isolate (Cao-Lormeau et al., 2014)	A549 (Human Adenocarcinomic human alveolar basal epithelial cell)	ZIKV induces mitochondrial apoptosis 48 h.p.i. by activating caspase 9 and 3.	Apoptosis is detected when the viral progeny reaches the peak.	[81]
MR766	HuH7 (Human hepatoma cell lines) and BCLX^KO^ HuH7 cells	MCL1 expression decreases in cells infected with ZIKV while BCLXL expression is not affected. BCLXL down-regulation induces cell apoptosis.	Decreased MCL1 expression during ZIKV infection promotes viral replication in vitro.	[86]
H/PF/2013 MR766 and Brazil 2015 BeH819015 (molecular clones)	A549, U251MG (derived from a human malignant glioblastoma), HEK293 (Human embryonic kidney 293 cells)	ZIKV infection leads to mitochondrial apoptosis when most of the ZIKV progeny is released by the infected cells. (48 h.p.i),	ZIKV delays apoptosis in infected cells and confers protection against exogenous apoptosis induced by either intrinsic or extrinsic pathways.	[87]
PLCal ZV: Asian strain isolated in 2013 in Thailand (Ellison et al., 2016) PRVABC-59: Asian strain isolated in Puerto Rico in 2015 (Lanciotti et al., 2016)	HFAs (Human fetal astrocytes), A549	Late apoptosis was induced in ZIKV HFA infected cells. Under 50% of the infected HFAs exhibited apoptosis compared to more than 90% for A549 5 d.p.i. This indicates that HFAs are remarkably resistant to apoptosis induced by ZIKV.	Although HFAs have a strong antiviral response, it has been shown that they keep excreting ZIKV for up to 28 d.p.i.	[88]
MR766 (molecular clone) PRVABC-59	Primary human Sertoli cells	Less than 10% of Sertoli PRVABC59 infected cells are apoptotic versus around 70% of A549 infected cells at 72 h.p.i. These percentages are half as low when cells are infected with the MR766 strain. The low level of ZIKV-induced apoptosis detected in Sertoli cells explains the persistence of both American and African ZIKV strains in these cells.	The limited percentage of apoptosis observed in ZIKV-infected Sertoli cells allows the virus to replicate furthermore. The peak of viremia was detected between 3 and 4 d.p.i.	[89]
r-MRV (recombinant MR766 strain) PRVABC-59	HTR-8 cells (Human immortalized first-trimester placental trophoblast cells), JEG-3 and JAR (choriocarcinoma-derived third-trimester placental trophoblast cell lines)	Apoptosis was induced in all three cell lines at 48 h.p.i. CHOP upregulation and nuclear translocation were observed 24 h.p.i. Trophoblast induced-apoptosis involved activation of caspases, ER-Stress markers and most importantly JNK protein.	Apoptosis was strongly inhibited by the use of JNK inhibitors.	[90]
MR766, SZ01 (Asian strain, isolated in China in 2016)	hRPTEpiCs human renal proximal tubular epithelial cells	MR766 induced a higher degree of cell apoptosis (48 h.p.i.) compared to SZ01 (9 d.p.i.)	ZIKV persisted for more than 30 d.p.i within the hRPTEpiCs.	[91]

h.p.i.: hours post-infection. d.p.i.: days post-infection h.p.t.: hours post-transduction N.D.: not determined.

### 4.1. Is Early Apoptosis in Development Responsible for the Irreversible Damage Produced by ZIKV Infection?

Most of the studies carried out suggest that ZIKV elicits apoptosis in vitro, around 48- or 72-h post-infection in cultured cell models. We and others have thus evidenced the mitochondrial events typical of PCD by apoptosis and the implication of BAX and caspases in fibroblast HT1080, in epithelial cell lines such as A549, HEK 293 [81,94], in glioblastoma astrocytoma U-251 MG [95], and in neuroblastoma cell line SH-SY5 [96] and neural crest cells PC-12 [85].

Cells that may be key players in the unique pathogenic process of ZIKV, which depends on maternal–fetal transmission, blood–brain barrier crossing, or important points in the neurodevelopment, were specifically studied.

One of the main issues raised by the CZS observed during the last outbreaks was how ZIKV spread into the fetal compartment early in gestation and whether apoptosis could be involved in the process. The mechanisms of crossing the uterine–placental interface (UPI) by ZIKV have been extensively studied and reviewed. Many cell types of the UPI support ZIKV replication. Placental infection, associated cytopathic effects and injury were characterized ex vivo, in villus explants from the first-trimester of pregnancy, in primary cells isolated from placental tissues, and in several relevant cell-lines [97,98,99,100].

Santara et al. found that in the first-trimester placenta, infected trophoblasts under severe ER stress were being destroyed, particularly through direct killing by maternal decidual natural killer cells (dNK) [101]. Thus, Muthuraj et al. showed that human immortalized placental trophoblasts and human choriocarcinoma-derived cell lines responded to infection by implementing an UPR-dependent apoptosis, with activation of JNK and that this feature could contribute in the dissemination of ZIKV from mother to fetus [90]. As above-mentioned (Section 2.2), virus-induced apoptosis may be the result of ER stress and unresolved UPR. During ZIKV infection, not all PERK, ATF6, and IRE1 pathways of the UPR are activated in the same way in all models [102,103]. It was found that ZIKV was actually able to control these responses during infection, acting de facto on the downstream UPR-dependent apoptosis. Thus, the ATF6 pathway appears to be little or not active, depending on the model and potentially inhibited during ZIKV infection [87,104,105,106]. If it is admitted that the IRE-1 pathway of UPR could activate apoptosis through JNK phosphorylation [107], an important factor in UPR-dependent-apoptosis is the transcription factor CCAAT-enhancer-binding protein (C/EBP) homologous protein (CHOP), whose expression is directly linked to the PERK pathway [108]. However, if we and others have noticed a significant increase in the transcriptional expression of CHOP [104], there is a lack of information concerning the CHOP protein presence in ZIKV infected cells. Thus, in the A549 model, we showed that the CHOP factor and its pro-apoptotic translational program were not induced [92]. Assessment of its role in the virally induced UPR-dependent apoptosis is therefore difficult. Furthermore, the control that the virus may exert over this factor instead suggests that ZIKV deploys strategies to inhibit or delay apoptosis.

To complete the available data on the mechanisms of maternal–fetal transmission, it is also worth mentioning that a pro-inflammatory context, likely to alter the physical placental barrier function, a cell-to-cell infection process, and a Trojan horse role for infected Hofbauer cells would be the major causes of viral dissemination through the placenta and then to the fetus. It is therefore interesting to note that primary Hofbauer cells have been shown to be resistant to virus-induced cell death [109]. Furthermore, in contrast to the African strains, the Asian-American epidemic strains showed more frequent replication in Hofbauer cells, increased dissemination, and longer viremia, but with lower viral titers. It was then proposed that the African strains, which were found to be more cytotoxic, might induce a better immune response and less accessibility to crossing the maternal–fetal barrier [110]. The timing or extent of apoptosis in placental tissue could be an example of the dual action of ZIKV on cell death to improve its dissemination strategy.

To gain an insight into the pathophysiology of ZIKV in neurodevelopment, infections have been conducted in neuronal models. Apoptosis was then shown to be induced in human neural progenitors (hNPC) and associated with P53 and cell cycle impairments [111]. In addition, in vitro models of hNPC derived from induced pluripotent stem cells (iPSC) were found to be susceptible to infection with mitosis abnormalities and cytopathic effects marked by caspase-3 activation [83].

Among the studies conducted ex vivo, sliced human fetal brain tissues from 14 to 21 weeks of gestation were infected with ZIKV. Activated caspase 3 and DNA fragmentation could be found in numerous infected and non-infected cells. Targeted cells were intermediate progenitor cells (IPCs) and post-mitotic neurons, confirming that ZIKV-induced neuropathogenesis could be the consequence of the disappearance of these cell populations [112].

Several in vivo study models support this assertion. In a mouse model of intracranial infection at different stages of embryonic development, co-immunoreactivity throughout the brain of cleaved caspase-3 and ZIKV are indicators of deleterious cell death [8]. In a recent study, oligodendrocyte loss in spinal cord and white matter has been linked to neurodevelopmental defects and perinatal ZIKV induced pathogenesis in mice [113]. In another chick embryo model, virus-induced apoptosis of cranial neural crest cells was shown to be responsible for aberrations in cranial osteogenesis and to lead to birth defects [114]. Finally, studies in pregnant non-human primate infection models revealed that neuroprogenitor apoptosis followed placental and fetal vascular compromise [115].

It should be noted that the study of ZIKV-induced patho-neuro-physiology has greatly benefited from the development of complex neurosphere and brain organoid models. Organoids are 3D cell cultures of self-organized progenitor cells that mimic the tissue architecture and development [116]. Several studies on these models have shown a size reduction in ZIKV infected neurospheres and brain organoids and a depletion in neural progenitors [75,78,110].

Transcriptome analyses performed on infected hNPCs, SH-SY5Y neuroblastoma cells or brain organoids revealed that ZIKV modified the expression of several factors related to apoptosis, neuronal development, and differentiation [75,84,96,117,118]. ZIKV infection resulted in increased expression of BAX, BID, and APAF1 in SH-SY5Y [96,117]. Caspase-3 gene was overexpressed in hNPCs [84] such as BAX, Bcl-2, and genes involved in tumor necrosis factor-related apoptosis-inducing ligand (TRAIL) signaling and TRAIL-mediated apoptosis such as death receptors DR4 and DR5 [119]. ZIKV infection could increase cell susceptibility to extrinsic death, suggesting that a bystander cytotoxic effect in developing brain tissue should not be minimized. Among the molecular mechanisms that could lead to cell death, it has been shown that ZIKV was able to induce p53 activation and to inhibit the mTOR pathway with an early switch from glycolysis to oxidative phosphorylation [120]. The changes in cellular metabolism initiated by the virus at a vulnerable period of neuronal development would then be responsible for the defects in differentiation of immature neural stem cells and their exhaustion. Another study showed that an epidemic strain of ZIKV provoked cell cycle arrest in neural stem cells by activating the p53-p21 signaling pathway [121]. This suggests that in addition to cell death, ZIKV-induced neuropathogenesis could also be due to proliferation and differentiation failures.

As a first look at all the data linking cell death and post-infection pathophysiology, it seems that a consensus has emerged on the capacity of ZIKV to induce an excess of apoptosis too soon during the particular situation of gestation and fetal development. This cell death would occur at the level of placental cells, which would facilitate the dissemination of the virus to the fetus. Apoptosis would then concern the neuronal progenitors and astrocytes. It would be responsible for a depletion of neuronal populations and for definitive damage during the development of the fetal brain.

### 4.2. Is Delayed and Impaired Apoptosis Responsible for ZIKV Persistence and Unusual Transmission Pathways?

A delayed apoptosis controlled by ZIKV was supported by mathematical models [122] and experimental approaches. By comparing studies describing the onset of in vitro virus-induced apoptosis in different cell models (Table 1), it appears that ZIKV triggers a delay of this cellular response and that this delay is to its own benefits. In in vitro infection models of epithelial cells, the first cytopathic effects occur after the completion of the viral cycle and the release of the viral progeny [81,87]. This timing is very different from that produced by infection with other arboviruses of the alphavirus family such as Chikungunya virus (CHIKV) or Ross River virus (RRV) [87,123]. At equivalent multiplicities of infection in the same cell types, the latter induced apoptosis as early as 6h post-infection, whereas with ZIKV, the first signs of PCD were only visible 48 h post-infection. In their studies, Limonta and colleagues argued that astrocytes were infected by ZIKV with reduced cytopathic effects including apoptosis, probably through FGF-2 upregulation during infection [88,93]. Therefore, they hypothesized that a ZIKV-driven anti-apoptotic activity was correlated with long-term infection and with the persistence of replicating virus in astrocytes.

A body of evidence points to the ability of ZIKV to manipulate cell death in order to limit the antiviral action produced by the cell auto-destruction. Thus, several studies have shown that apoptosis has an antiviral effect on ZIKV. Addition of the pan-caspase inhibitor, zVAD-FMK during infection leads to an intracellular increase in viral RNA [83]. The use of inhibitors of the anti-apoptotic Bcl-2 family proteins, triggered an earlier apoptosis in infected cells by ZIKV [86]. In the same publication, Suzuki and colleagues reported that HuH7 cells that were knocked out for the anti-apoptotic gene BclXL underwent an accelerated apoptosis with a suppressed viral dissemination. This was observed with ZIKV and other flaviviruses such as DENV and JEV. BclXL gene suppression led to a reduction in the viral loads in cells and animal models. Last but not least, this apoptosis remediation also increased animal survival in JEV infected mouse models. Suzuki and co-authors concluded from their study that delayed apoptosis was associated with high viral pathogenicity, whereas early apoptosis combined with accelerated efferocytosis inhibited viral spread in the body and limited pathogenicity.

In the case of ZIKV infection and its unique pathogenesis with neurological complications, the role of anti-apoptotic factors and the control of their expression or activity by the virus have been examined. A clinical study of cases of fatal microcephaly found that the parenchyma of infected newborns had a two-fold increase in the anti-apoptotic Bcl-2 protein. This feature appears to be specific to CZS as microcephalies of other origin do not have variation in Bcl-2 expression [124]. Based on the observation that ZIKV seemed to be able to counteract apoptosis, our team had the idea of testing the protection that the virus could offer to cells against an exogenously induced apoptosis. Surprisingly, cell death hallmarks could not be observed in A549 cells when apoptosis was artificially induced two hours after infection with ZIKV. Inhibition of cell death in the presence of ZIKV was achieved whether the induction of apoptosis was extrinsic, with TNF-alpha, or intrinsic, with etoposide or blasticidin. This protection from exogenous apoptosis was potentially dependent on the presence of ZIKV non-structural proteins [87]. In addition, another study indicates that double-stranded RNA (Poly I:C) induced apoptosis is reduced by overexpressing the ZIKV NS2B/3 non-structural proteins [82].

We investigated which protagonists of apoptosis ZIKV was able to act upon and also observed that the amount of anti-apoptotic protein Bcl-2 was increased during in vitro infection. A privileged role for the Bcl-2-family of anti-apoptotics was confirmed by the use of the inhibitor ABT-737, which abrogated ZIKV-mediated protection, led to restoration of apoptosis, and reduced viral infection [87]. As previously mentioned, we also found that ZIKV was able to subvert the CHOP pro-apoptotic program and thus override ER-dependent apoptosis [92].

This viral ability to inhibit one of the main cellular defense responses to infection legitimately raises the question of its effect on the outcome of infection and, if unsuccessful, the possibility that the virus will not be properly eliminated. There is an extensive literature on the strategies developed by infectious agents to manipulate apoptosis and the link between impaired apoptosis, deficiencies in viral clearance, and viral persistence in privileged niches [125,126,127,128]. Numerous studies on the mechanisms and issues of ZIKV infection have revealed that the virus can indeed persist for a long time after the primary infection.

Initially puzzling to the scientific community, a first case of sexual transmission of ZIKV was reported in 2011 [129]. The confirmation that this route of transmission was far from anecdotal, and observed with both Asian and African strains of ZIKV raised the possibility that ZIKA could persist in the male reproductive organs [10]. Indeed, viral RNA have been detected in semen for up to six months post infection, suggesting that men may therefore act as potential reservoirs of ZIKV [130]. Reproductive tract and testis certainly offer a suitable immune-privileged environment for ZIKV persistence.

High-level and persistent viruria could also be observed for up to 15 days after the onset of infection symptoms. Several cell types of the infected renal tissue such as glomerular cells or renal proximal tubular epithelial cells (hRPTEpiCs) may be susceptible as reservoirs for long-term excretion of ZIKV in urine [91,131].

The maintenance of viral replication in these different cells, whether or not associated with their survival and incomplete apoptosis, deserves further investigation.

Then, the first clinical indications that the virus might also persist in the CNS were given when worrying cases of neurological complications were reported in infants a long time after their birth and their in-utero exposure to ZIKV [132,133].

In conclusion, a body of evidence indicates that ZIKV manipulates apoptosis, which is an adverse response of the cell to virus multiplication. This ability could account for the persistence of the virus in immunoprivileged niches. Inhibited or delayed apoptosis and viral persistence could influence pathogenesis and could explain the unique features of ZIKV infection. These main singularities are the non-vectorial routes of transmission and the ability to induce neuropathogenesis long after infection.

### 4.3. Apoptosis in the Mosquito Vectors

In mosquito cells, arbovirus infections rarely cause cytopathic effects, and the limited data currently available assessing the role of apoptosis in vector competence suggests that apoptosis is detrimental to the virus. Virus/vector interactions appear to have co-evolved by limiting apoptosis, probably to an initial regulation by non-retroviral integration RNA virus sequences (NIRVS) and the piRNA antiviral pathway [134]. In invertebrate cells infected with Zika virus, very few, if any, cytopathic effects are observed [135]. Furthermore, it seems that sfRNA from ZIKV could inhibit apoptosis whereas sfRNA mutants showed increased TUNEL positive cells in-vivo. Adding caspase inhibitors rescued a ‘wild type’ virus transmission rate in a sfRNA mutant, showing that apoptosis decreases the transmission rate in mosquitoes [136]. This confirms previous studies on arboviruses and apoptosis in mosquitoes [137].

Infection with ZIKV leads, in particular, to a reprogramming of cellular glucose metabolism in a human cell model by enhancing ATP production via the use of the tricarboxylic acid cycle [26]. In contrast, during infection of mosquitoes’ cells, ZIKV increased the use of glucose through the pentose phosphate pathway. Thus, this differential reprogramming of glucose metabolism changes the cellular ratio of AMP/ATP, which leads to the differential status of the AMPK phosphorylation level between mosquitoes and humans during ZIKV infection. The AMPK activation in human cells contributes to caspase-mediated cell death, and conversely, low activation of AMPK in mosquitoes’ cells prevents apoptosis [26].

All of these different apoptosis control mechanisms are important for vector competence, and thus virus spread between mosquitoes and humans.

## 5. Therapeutics Related to ZIKV, A Role for Apoptosis?

In addition to preventive measures, which mainly involve personal protection against mosquito bites, it is essential to respond to the Zika threat by developing treatments and vaccines. Currently, we have neither one nor the other, although a vaccine candidate using mRNA technology has recently shown promising results in mice [138]. The development of anti-ZIKV drugs must take into consideration several specificities such as the passage of the virus in immune-privileged sites (central nervous system, placenta, gonads) and it must be adapted for the particularly fragile target of pregnant women or women planning a pregnancy.

One approach to containing the infection is to fight the virus early by acting on the virus particle itself or at a particular stage of the viral cycle (entry, replication). Unfortunately, although many studies have been carried out and have proposed molecules with promising effects in vitro, no validated treatment has yet been improved [139,140,141].

Alternative strategies focus on enhancing the natural antiviral responses of host cells by repurposing already known active therapeutic molecules. Trials using the proteasome inhibitor bortezomib have demonstrated antiviral activity in in vivo models and have provided some hope [142,143]. The advantage of targeting host responses is to limit the risk of the emergence of treatment-resistant viral variants, as has been observed with nucleotide analogues [144,145].

### 5.1. Antiviral Treatment through Apoptosis Remediation

Based on the observation that many viruses, and this is the case of ZIKV, manipulate the cell death response and that this capacity contributes to infectious processes (outcome, pathogenesis, viral persistence), therapeutic options using apoptosis remedial action have been explored. The underlying idea is that restoring apoptosis can improve or accelerate the resolution of infection and free the body from potential virus persistence [146]. Apoptosis sensitizers such as inhibitors of the Bcl-2 family or molecules mimicking suppressors of apoptosis inhibitors (synthetic SMAC/DIABLO proteins) have thus been tested as antiviral candidates [147]. From this theory of a ‘functional reactivation of apoptosis’ during infection, an antiviral strategy caused a sensation in 2011. By combining the double-stranded RNA recognition domain of the PKR protein and the caspase interaction domain (CARD) of the Apaf1 protein, the technology called double-stranded RNA (dsRNA) activated caspase oligomerizer (DRACO) showed broad-spectrum antiviral activity and lack of toxicity in murine, human, and in vivo non-human primate cells [148]. It resulted in an 80% reduction in mortality in a mouse model of H1N1 A/PR/8/34 infection. Following the same idea, the antiviral therapeutic potential of molecules known to act by reactivating apoptosis in cancer pathologies was tested on ZIKV infection, with the project of a possible drug repositioning. One study successfully evaluated the anti-ZIKV activity of the anti-cancer obatoclax or GX15-070, a pan-blocker of the Bcl-2 family of proteins [149]. It should be noted that the use of inhibitors of Bcl-2 family proteins had previously been validated for their capacity to inhibit several viruses other than ZIKV, in in vitro and in vivo studies [147].

### 5.2. ZIKV as Oncolytic Virotherapy

**Figure 4:**
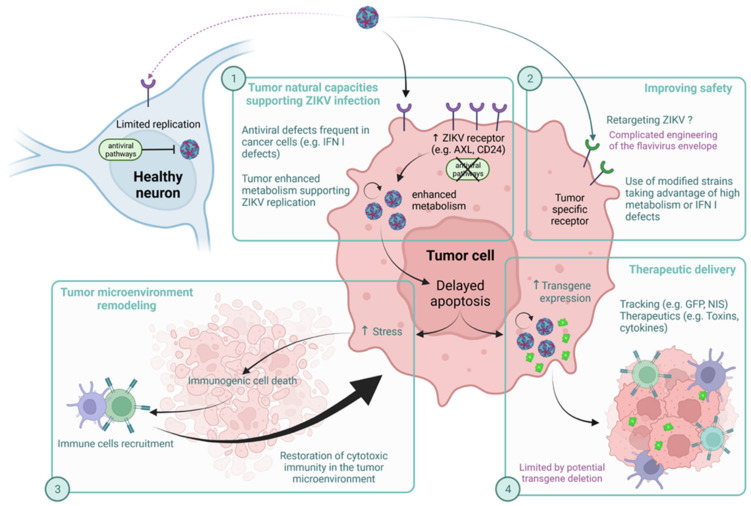
Zika as an oncolytic agent, pros and cons. ZIKV has natural tropism for neurons but its replication is counteracted by antiviral pathways. Tumor cells overexpress receptors for viral entry, have a high metabolism that efficiently supports ZIKV replication, and have frequent defects in antiviral pathways. These natural capacities enhance viral infection (1). To further improve its tropism toward the tumor, ZIKV could be modified to specifically target tumor cell receptors or to be more dependent on the tumor’s singular properties (2). Delayed apoptosis induced by ZIKV replication allows for a more immunogenic cell death, which can turn an immunosuppressive tumor microenvironment into an immunoactivating one (3). Delayed apoptosis also promotes transgene expression allowing for more efficient delivery of therapeutics (4).

Interestingly, ZIKV has been considered as an oncolytic candidate, notably for therapy of brain cancer [150]. The modes of action of ZIKV on the tumor and the advantages and disadvantages of using this virus, which may be genetically modified, are summarized in Figure 4. Briefly, oncolytic viruses are replicative viruses capable of specific replication in cancer cells. These biological agents have cancer therapeutics potential due to their ability to induce selective tumor cell death through direct cytopathic effects. They induce an immunogenic cell death similar to apoptosis, which releases cell debris and viral antigens. Dendritic cells from the microenvironment will then allow the immune system to be educated against the tumor [151,152]. Countering the frequent immune escape phenomena induced by the tumor microenvironment is therefore one of the major goals of new anti-cancer therapies. Showing the important potential of this type of treatment, the FDA already approved the use of Talimogene Laherparepvec (T-VEC), a modified herpes simplex virus type I, for metastatic melanoma management [151].

As aforementioned, ZIKV has a privileged tropism for neuronal cells in which it produces cytopathic effects [83,111]. This specificity is due to the high level of neuronal expression of the main known ZIKV membrane receptors such as AXL or CD24. Interestingly, these receptors are overexpressed in cancer cells. Due to this ability, ZIKV has been proposed as a promoter of tumor mass reduction for some brain cancers (e.g., glioblastoma multiforme (GBM) and neuroblastoma). Indeed, in 2017, a selective oncolytic activity was shown against glioma stem cells (GSCs) [153], which are therapy-resistant self-renewing tumor precursor cells responsible for the local recurrence of cancer. ZIKV infection in these cells leads to a loss of self-renewal and proliferation due to increased apoptosis, whereas no detrimental effects were observed on normal cells. However, this feature is not a general property of neurotropic flaviviruses, since WNV causes the cell death of GSCs and normal cells. In order to confirm these promising results and show the usefulness of ZIKV as a therapy to treat brain cancer, the authors used a mouse model of glioma. Mice receiving ZIKV injection showed prolonged survival in comparison to mock-infected mice due to extensive tumor cell death.

Consistent with these results, intracerebral injection of a live attenuated Zika virus (ZIKV-LAV) in a mouse model of human GBM significantly reduced intracerebral tumor growth and prolonged animal survival by selectively killing SOX2+ GSCs within the tumor [154]. Recently, the same team reported some details on the mechanism underlying ZIKV oncolytic activity against GSCs [155]. They found out that SOX2 expression in GSCs leads to downregulation of the IFN signaling pathway, this being essential for ZIKV infection and replication in GSCs. Indeed, SOX2 is a core regulator of antiviral response and apoptosis, and is found to be highly expressed in GSCs, which partly explains the preferential lytic effect on GSCs. Moreover, SOX2 has also been associated with the regulation of Integrin ɑV expression. Integrin ɑV forms heterodimers, notably with Integrin β5, whose blockade with specific antibodies has led to reduced ZIKV-induced oncolytic effects on GSCs. Thus, the SOX2–Integrin ɑVβ5 axis seems to be crucial for oncolytic activity against GSCs, and therefore GBM treatment.

It has also been shown that ZIKV oncolytic activity requires CD8+ T cell recruitment to the tumor microenvironment, as the survival benefits are lost if CD8+ T cells are depleted [156]. ZIKV infection enhances immune infiltration, which is favorable for combination with an anti-PD1, an immune checkpoint inhibitor to remove microenvironment induced T-anergy. Moreover, this education of CD8 by ZIKV induced tumor cell death persists over time. Hence, they protect mice against syngeneic tumor rechallenge. Additionally, neuroblastoma cells’ permissiveness for Zika virus has been reported [157]. In neuroblastoma cells, ZIKV-induced cytopathic effects lead to a decrease in tumor cell viability. Nevertheless, not all neuroblastoma cell lines were sensitive to ZIKV oncolytic activity. It was shown that CD24 expression, a receptor expressed on the surface of metabolically active cells including cancer cells [158] is essential to ZIKV oncolytic activity [157]. Restoring CD24 (SK-N-AS) expression in a low-permissive neuroblastoma cell line restores ZIKV oncolytic activity, unlike the CD24-deficient cell line. CD24 is rather a brake on apoptosis, which could promote ZIKV replication in metabolically active cells and consequently induce a better immunogenicity [152,158]. Therefore, ZIKV seems to be a powerful tool for the treatment of brain cancers that are of critical concern. As raised by several teams [151,154,155], the use of ZIKV as oncolytic therapy can only be considered if the safety conditions are met. Data currently available from these studies indicate that viral RNA remained localized to the tumor until two weeks after treatment, showing the absence of viral spread following injection [153].

However, as discussed above, ZIKV can persist in the body and has a broad tissue tropism. There is therefore concern that it may affect several organs other than the target tumor. These issues could be circumvented by improving ZIKV specificity to tumor cells. Indeed, receptors essential for ZIKV entry such as AXL and CD24 that are overexpressed in glioblastoma and neuroblastoma cancer cells, respectively, are also present on many other cell types [158,159]. Thus, addressing ZIKV to a specific receptor on tumor cells would reduce its entry into healthy cells. Technically, modifications can be made to the viral genome to fine-tune its oncolytic capabilities. However, engineering the viral envelope to control the interaction of the viral particle with the cell surface is difficult to design and implement. If retargeting is not possible or not sufficient, another option is to increase the dependence of the virus on the singular properties of the tumor cells. Indeed, it would be possible to make the virus more conditional on the specific enhanced metabolism of the tumor cell, as is the case for the vaccinia virus TK-RR- [160] (or the measles virus vaccinal strain [161]. Genetically-modified ZIKV strains should then be envisaged to further improve the safety of a ZIKV based oncolytic virotherapy [153,154].

Finally, and regarding the ambiguity revealed by our literature review on the ability of ZIKV to promote apoptosis too early or too late, it would appear that delayed or attenuated cell death is not a pitfall, but may be an advantage in oncolytic therapy. Thus, cytolytic effects on infected cells, by being delayed, will promote immunogenicity with a progressive and sustained recruitment of immune cells [156,162]. This mechanism is decisive in overcoming immunosuppression induced by the tumor environment, one of the main strengths of virotherapy. It should also be noted that the possibilities of manipulating oncolytic viruses have led to their proposal as vectors for therapeutic agents such as T-VEC, which encodes for granulocyte-macrophage colony-stimulating factor to increase its immunostimulant properties [163]. In this context, a virus with persistent replication still has an advantage in that it can increase the production of the therapeutic protein over time. In addition, delayed apoptosis becomes an advantage for this vectorization process. Whether strictly for oncolytic purposes or as a genetic backbone for the delivery of a recombinant therapeutic agent, this ability of ZIKV to induce delayed cell death makes it a potentially good vector for antitumor therapy. However, these beneficial effects may be mitigated by the ability of ZIKV to persist in some tissues, with detrimental effects on patients. Despite this, studies have shown that, in dogs with glioblastoma, the use of a Brazilian ZIKV reduced tumor size and did not induce clinical side effects [164]. Further research is needed to decipher the potential of ZIKV as an oncolytic treatment for brain cancers [153,154].

## 6. Discussion and Conclusions

In the last decade, a series of epidemics has put global human health at risk. This pressure is due to the threat of mainly viral pathogens that do not spare any region, regardless of the economic levels or medical capacities of countries. In this context, better knowledge of infectious agents, their mechanisms of dissemination/transmission in the environment, and their interactions with their hosts, particularly humans, is essential. This is necessary to face the risks of viral emergence or re-emergence as well as current and future epidemics and pandemics. Understanding the mechanics of host–pathogen interactions at the cellular and molecular levels is also essential for the development of preventive and/or therapeutic strategies. Among the cellular and molecular responses that are crucial for eliminating the pathogen, but that may also be involved in pathological processes is cell death by apoptosis. Just as humans evaluate the benefits and risks of an action, the cell must make a choice between survival and self-killing to resolve an infection. The decision to commit suicide is based on a precise control of pro-apoptotic and anti-apoptotic factors. The process of ZIKV infection seems to be a wonderful example of manipulation of this pro-death or survival balance of the host cell.

**Figure 5:**
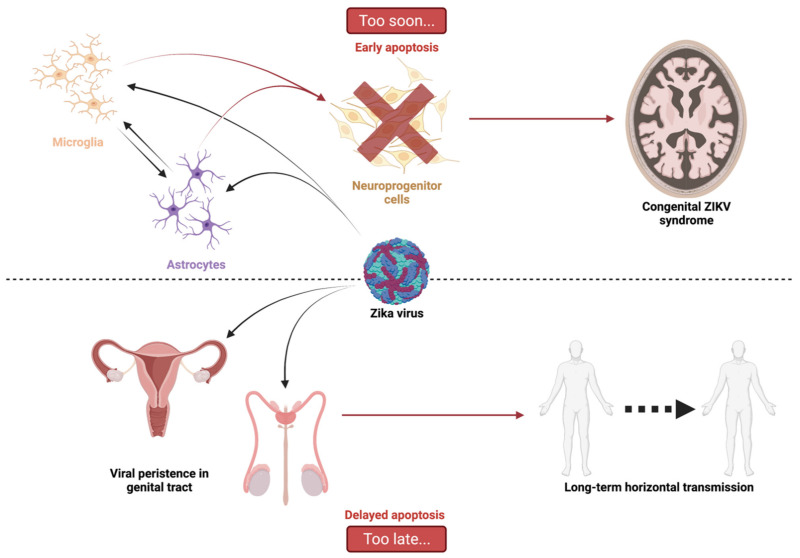
Too soon or too late, graphical abstract. ZIKV-related apoptosis is ambivalent. On one hand, the virus transmission to the central nervous system via microglia and astrocytes induces early apoptosis of neuroprogenitor cells. This leads to a set of symptoms and defects grouped under the term of ZIKV congenital syndrome. On the other hand, ZIKV has been shown to persist for a while in infected organisms due to delayed apoptosis. Persisting in the genital tract could lead to sexual transmission that is unusual for an arbovirus.

ZIKV, similar to other flaviviruses (such as JEV, DENV or WNV), is capable of inducing apoptosis in various types of infected cells. Therefore, an excess of placental cell apoptosis, early in gestation, seems to be one of the main causes of horizontal maternal–fetal transmission. Dissemination of the virus into fetal tissues, infection of the brain, and early apoptosis of neuronal progenitors seem to be involved in Zika pathology and SCZ development (Figure 5).

Conversely, cell death by apoptosis has been shown to occur late in many cell types. This delay in the onset of cell death involves anti-apoptotic proteins such as Bcl-2, whose stability and half-life are increased by a mechanism that remains to be defined. It also involves a defect in the induction of the expression of pro-apoptotic factors (BIM, PUMA, NOXA...) that are cruelly lacking to tip the balance. This defect is in part due to the ability of ZIKV to ‘hack’ the communication pathway between ER stress, UPR, and apoptosis by inhibiting the CHOP factor. Other studies have described that paraptosis is sometimes induced by ZIKV, with hyper vacuolation of cells without caspase activation. This could be in place of apoptotic death and apparently allows the virus to replicate more abundantly in the cell [165]. This confirms that apoptosis is detrimental to the virus and this raises the question of the impact of delayed apoptosis on pathophysiological processes and viral persistence following ZIKV infection. A persistence capacity of ZIKV is established. The virus, which remains in the genital tract and is secreted for a long time in the semen, is the cause of sexual transmission (Figure 5). In addition to the complications observed in newborns, the evidence of neuropathologies in children after birth [132] raises questions about the mechanisms involved long after the acute infection. Recent work also suggests that ZIKV exposure may contribute to the development of neurodegenerative pathologies in adults in the longer term [103,166]. Of note, data on microcephalic brains of ZIKV-infected neonates have shown an increase in Bcl-2 protein, opening up the hypothesis of viral persistence mediated by overexpression of anti-apoptotic proteins [124]. Taken together, these insights into ZIKV infection confirm the interest in understanding the mechanisms of apoptosis control. This understanding is important for the development of antiviral therapies based on the restoration of the ability of infected cells to die. It could also be useful in the fight against late-onset forms of Zika. This understanding is ultimately important for considering the use of this singular virus in oncolytic virotherapy.

## Abbreviations

ZIKV	Zika virus
PCD	Programmed cell death
CNS	Central Nervous System
ER	Endoplasmic Reticulum
MOMP	Mitochondrial outer membrane permeabilization
APAF-1	Apoptosis protease activating factor 1
PARP	Poly ADP-ribose polymerase
DISC	Death inducing signaling complex
UPR	Unresolved unfolded protein response
CHOP	C/EBP HOmologous Protein
BHV	Bovine herpesvirus 1
DENV	Dengue virus
WNV	West Nile virus
JEV	Japanese encephalitis virus
HSV	Herpes simplex virus HSV
MAVS	Mitochondrial antiviral-signaling protein
WHO	World Health Organization
CDC	Center for disease control
CZS	Congenital Zika syndrome
sfRNA	Small flavivirus RNA
CHIKV	Chikungunya virus
RRV	Ross River virus
